# Hemin activation abrogates *Mycoplasma hyorhinis* replication in chronically infected prostate cancer cells via heme oxygenase‐1 induction

**DOI:** 10.1002/2211-5463.13271

**Published:** 2021-09-09

**Authors:** Hanxia Huang, Alena Dabrazhynetskaya, Jacob Pluznik, Jiwen Zheng, Yong Wu, Vladimir Chizhikov, Paul W. Buehler, Kenneth M. Yamada, Subhash Dhawan

**Affiliations:** ^1^ Center for Biologics Evaluation and Research, Food and Drug Administration Silver Spring MD USA; ^2^ Office of Science and Engineering Laboratories Center for Devices and Radiological Health, Food and Drug Administration Silver Spring MD USA; ^3^ National Institute of Dental and Craniofacial Research National Institutes of Health Bethesda MD USA; ^4^ Present address: Department of Pathology Center for Blood Oxygen Transport and Hemostasis Department of Pediatrics University of Maryland School of Medicine Baltimore MD USA; ^5^ Present address: Retired Senior FDA Research & Regulatory Scientist 9890 Washingtonian Blvd., #703 Gaithersburg MD 20878 USA

**Keywords:** heme oxygenase‐1, host defense, infectious disease, innate immunity, *Mycoplasma hyorhinis*, prostate cancer

## Abstract

*Mycoplasma hyorhinis* (*M. hyorhinis*) lacks a cell wall and resists multiple antibiotics. We describe here the striking > 90% inhibitory effect of hemin, a natural inducer of the cytoprotective enzyme heme oxygenase‐1 (HO‐1), on *M. hyorhinis* replication in chronically infected LNCaP prostate cancer cells. The role of HO‐1 in interrupting *M. hyorhinis* replication was confirmed by HO‐1‐specific siRNA suppression of hemin‐induced HO‐1 protein expression, which increased intracellular *M. hyorhinis* DNA levels in LNCaP cells. Proteomic analysis and transmission electron microscopy of hemin‐treated cells confirmed the complete absence of *M. hyorhinis* proteins and intact microorganisms, respectively, strongly supporting these findings. Our study is the first to our knowledge suggesting therapeutic potential for activated HO‐1 in cellular innate responses against mycoplasma infection.

## Graphical abstract

This study demonstrates inhibition of antibiotic‐resistant mycoplasma *M. hyorhinis* replication in human prostate cancer cells by the inducible cytoprotective enzyme heme oxygenase‐1 (HO‐1). Hemin induces mRNA and protein for this ubiquitous HO‐1 component of innate cellular immunity. Hemin is the active component of an FDA‐approved drug for another indication and could potentially provide a therapeutic modality for treating mycoplasma infection.

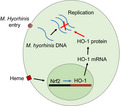

**Abbreviations**
HO‐1: heme oxygenase‐1
M. hyorhinis: Mycoplasma hyorhinis
TEM: transmission electron microscopy

Mycoplasmas belong to the family of one of the smallest prokaryotes that can colonize human and animal tissues for an extended period of time, often without any apparent pathology. The fastidious nature of mycoplasmas often poses challenges for isolation and identification of the microorganism from affected tissues to select an efficient treatment of disease conditions [1]. As a result, many mycoplasma infections remain unidentified and untreated. Due to their small size and the absence of a rigid cell wall around their cell membranes, these microorganisms are generally resistant to many antibiotics [2], therefore posing a serious clinical challenge for proper treatment of infected individuals. Mycoplasmas are well known as laboratory contaminant of various cells and tissue cultures [3] that can alter experimental results.

*Mycoplasma hyorhinis (M. hyorhinis)*, first identified in swine in 1962, can cause respiratory tract infections, arthritis, and inflammation of the abdominal cavity [4]. Recent studies indicate a potential link between mycoplasma infection and several human cancers, such as prostate, gastric, and ovarian cancer [5, 6, 7, 8, 9, 10]. In particular, *M. hyorhinis* infection with its increased antibody titer and elevated prostate‐specific antigen (PSA) levels is reported to result in malignant changes in benign human prostate cells [11, 12]. An increased rate of seropositivity to mycoplasma, especially *M. hyorhinis*, in men with prostate cancer (52%) compared to those with benign prostate hyperplasia (36%) further suggests a link between *M. hyorhinis* infection of normal prostatic tissue and risk of a prostate cancer [13].

Mycoplasmas are self‐replicating organisms with the smallest genomes, containing a total of about 500–1000 genes [14]. Due to their extremely basic genome and metabolic dependence, mycoplasmas function as parasites for their survival [15]. Most mycoplasmas reside on the surface of cells; however, certain species of mycoplasma, including *M*. pneumonia and *M. hyorhinis*, invade cells and cause disease or promote disease progression [16, 17, 18]. Invading mycoplasmas subvert host protective responses for replication and cellular transformation, impacting cellular functions for rapid disease progression.

Association of *M. hyorhinis* with various cancers pose a significant challenge to drug therapy. Since antibiotics are largely ineffective for treating intracellular mycoplasma infections, augmentation of the cellular defense response presents an alternative approach for targeting mycoplasma replication. Recent studies from our and other laboratories have demonstrated the involvement of inducible HO‐1 in the innate host defense mechanism against a wide variety of infections [19, 20, 21, 22, 23, 24, 25, 26, 27, 28, 29]. In the present study, we tested whether the induction of HO‐1 would also limit the replication of the prokaryote, *M. hyorhinis* in mycoplasma‐infected cells. We found that HO‐1 induction by its natural substrate hemin dramatically reduced *M. hyorhinis* replication in chronically infected LNCaP prostate cancer cells, further demonstrating a pivotal role for this endogenous cytoprotective enzyme in the host defense mechanism against invading pathogens.

## Materials and Methods

### Materials

LNCaP cells were obtained from the American Type Culture Collection (Manassas, VA) and were maintained at 37 °C in a humidified incubator with a 5% CO_2_/95% air atmosphere in RPMI 1640 supplemented with 10% FCS. These cells were subsequently found positive for *M. hyorhinis*. SYBR Green RT‐PCR MasterMix was obtained from Qiagen (Valencia, CA, USA). The FDA‐approved drug Panhematin^®^, containing hemin as the active component, was purchased from Lundbeck, Deerfield, IL (manufactured by APP Pharmaceuticals, Raleigh, NC, USA). Small interfering RNA (siRNA) targeting human HO‐1‐coding sequences (Hs_HMOX1_1 and Hs_HMOX_10) and AllStars Negative Control siRNA were from Qiagen. All other experimental components were of reagent grade.

### DNA extraction from cells

Total genomic DNA was extracted from LNCaP cells using the Qiagen DNA isolation kit (Qiagen, Valencia, CA, USA) according to the manufacturer’s protocol. The DNA pellet was resuspended in 30 μL of DNAse‐free distilled water and stored at −20 °C. The quality of DNA was assessed by Agilent Bioanalyzer (Santa Clara, CA, USA).

### Quantitative *M. hyorhinis* polymerase chain reaction (PCR)

Real‐time PCR amplification of *M. hyorhinis* in the total genomic DNA was performed using QuantiTect SYBR Green PCR kit (Qiagen) with HotStar Taq^®^ DNA polymerase and SYBR Green I fluorescent dye according to the manufacturer’s protocol, and universal mycoplasma primers 5′‐GGCGAATGGGTGAGTAACACG‐3′ (forward) and 5′‐GGATAACGCTTGCGACCTATG‐3′ (reverse) as described previously [30]. Briefly, each 50 μL reaction contained of 25 μL of 2X SYBR Green PCR Master Mix, ≤ 0.5 µg DNA template, 0.5 μm of forward primer, 0.5 μm of reverse primer. Amplification was performed in an Applied Biosystems 7500 real‐time PCR or Applied Biosystems QuantStudio 6 Flex system at the following conditions: 1st step—DNA denaturation and Taq polymerase activation at 95 °C for 15 min.; 2nd step—40 cycles of denaturation at 95 °C for 15 s., annealing at 60 °C for 30 s and elongation at 72 °C for 1 min; 3rd step—final elongation at 72 °C for 5 min; and 4th step—dissociation curve analyses for obtained PCR products. The number of *M. hyorhinis* genomic copies was calculated based on comparison of the Ct values with standard curves generated from the purified known copy numbers of *M. hyorhinis* DNA.

### Quantification of cell‐free *M. hyorhinis*

LNCaP cells chronically infected with *M. hyorhinis* were first cultured for 24 h in the absence or presence of 100 μm hemin followed by replacing the media with serum‐free media lacking hemin and then culturing cells for an additional 24 h. *M. hyorhinis* DNA was amplified directly from 5 μL of the culture supernatants without DNA extraction. The number of *M. hyorhinis* copies was calculated based on comparison of the Ct values with standard curves generated from *M. hyorhinis* DNA template (BTS‐7 strain) as described above. All qPCRs, including standards, were performed in duplicate.

### Determination of total cell number and cell viability

LNCaP cells cultured for 48 h in the absence or presence of antibiotics and hemin at the desired concentrations. Total number of cells were quantified microscopically, and the cell viability was determined by trypan blue exclusion test.

### Small interfering RNA (siRNA) transfection

Small interfering RNAs (siRNAs) targeting human HO‐1‐coding sequences (Hs_HMOX1_1 and Hs_HMOX_10) were purchased from Qiagen. Although the sequences of these siRNAs are proprietary for Qiagen, the corresponding target nucleotide sequence for siRNA Hs_HMOX1_1 (catalog number: SI00033089) is CACCAAGTTCAAGCAGCTCTA and that for siRNA Hs_HMOX1_10 (catalog number: SI04435354) is CAAGACTGCGTTCCTGCTCAA, respectively. AllStars Negative Control siRNA (Qiagen) was used as the nontargeted siRNA control. LNCaP cells were seeded in 6‐well culture plates for transfection and incubated with 50 nm control or HO‐1 siRNA for 6 h in serum‐free OPTI‐MEM media following the manufacturer’s transfection instructions using Lipofectamine® RNAiMAX (Invitrogen, Carlsbad, CA, USA). The cells were then treated with 100 μm hemin. The efficiency of HO‐1 knockdown was assessed by western blot analysis. The level of *M. hyorhinis* DNA was quantified by real‐time PCR using universal mycoplasma primers as described above.

### Protein extraction and western blot analysis

HO‐1 induction in LNCaP cells was determined by western blot. Briefly, cells cultured in 6‐well plates were pretreated for 24 h with various concentrations of hemin ranging from 0 to 100 μm, and HO‐1 induction was determined by western blot. Briefly, total cell protein extracts were prepared in modified RIPA buffer (50 mm Tris/HCl, 1% NP‐40, 0.25% deoxycholic acid, 150 mm NaCl, 1 mm EGTA, 1 mm sodium orthovanadate and 1 mm sodium fluoride with protease inhibitors (Roche Applied Science, Mannheim, Germany) and phosphatase inhibitors (Sigma‐Aldrich, St. Louis, MO, USA) and quantified using the Pierce BCA protein assay. Total protein (2.5–5 μg) was resoled on a 12% Tris‐glycine SDS/PAGE gel. After transfer to a PVDF membrane (Millipore, Burlington, MA, USA), immunoblots of whole cell lysates from untreated and hemin‐treated LNCaP cells were analyzed using a cocktail of mouse monoclonal anti‐HO‐1 IgG (Catalog # ADI‐OSA‐110; Enzo Life Sciences, Farmingdale, NY, USA) and polyclonal rabbit anti‐actin (Catalog # ab8227; abcam, Cambridge, MA, USA) antibodies. The blots were washed and then placed in a cocktail of secondary HRP‐conjugated anti‐mouse (Catalog # NA 931V) and anti‐rabbit IgG (Catalog #NA 934V; GE Healthcare, formerly Amersham) each diluted 1:2000 in 2% nonfat dry milk in Tris‐Tween‐buffered saline (TTBS) for two hours and rinsed 4 times for 10 min in TTBS. The HO‐1 protein (32 kDa) and actin (42 kDa) bands were visualized using the ECL® (enhanced chemiluminescence) detection system (GE Healthcare, Chicago, IL, USA), and exposed to X‐ray film.

### Proteomic analysis and mass spectroscopy

Proteomic analysis (2‐DIGE) and mass spectrometric identification of proteins from untreated and hemin‐treated LNCaP cells were performed by Applied Biomics, Inc. (Hayward, CA, USA) as described below.

### Sample preparation

LNCaP cells that were untreated (control) or treated for 48 h at 37 °C with 100 μm hemin were washed three times with cold PBS, gently scraped off the dish, centrifuged at 200 ***g***, and resuspended in 100 μL of 2‐D cell lysis buffer (Applied Biomics) and then stored at −80 °C until sending to Applied Biomics on dry ice for proteomics analysis. Briefly, 2‐D cell lysis buffer (30 mm Tris/HCl, pH 8.8, containing 7 M urea, 2 M thiourea and 4% CHAPS) was added to the collected cell pellets, sonicated on ice, followed by gentle shaking on a shaker for 30 min at room temperature. The total protein lysates were centrifuged at 25 000g for 30 min at 4 °C, and supernatants were collected. Protein assays were performed using the Bio‐Rad protein assay method. Lysate were diluted with the 2‐D cell lysis buffer to adjust the final protein concentration to 6 mg/mL.

### Minimal CyDye labeling

To 30 μg of protein lysate, 1.0 μL of diluted CyDye (1:5 diluted with DMF from 1 nmol/μL stock) was added and mixed thoroughly, and then, the tube was placed in the dark on ice for 30 min. 1.0 μL of 10 mm lysine was added to each of the samples and vortexed, and the reaction mixture was maintained in the dark on ice for an additional 15 min. Samples were labeled with Cy2, Cy3, and Cy5 dyes and diluted with equal volumes of 2‐D sample buffer (8 M urea, 4% CHAPS, 20 mg/mL DTT, 2% Pharmalytes and trace amount of bromophenol blue). 100 μL destreak solution and rehydration buffer (7 M urea, 2 M thiourea, 4% CHAPS, 20 mg/mL DTT, 1% Pharmalytes and trace amount of bromophenol blue) were added to a final volume of 250 μL for the 13 cm IPG strip. The sample mixture was thoroughly mixed, centrifuged to clarify the solution, and equal amounts (30 μg) of protein were loaded onto the strip holder.

### Isoelectric focusing and SDS/PAGE

After loading the labeled samples onto the strip holder, the 13 cm strip was placed facing downward, and 1 mL mineral oil was added on the top of the strip. The IEF was run in dark at 20 °C following the GE BioSciences protocol. At the end of the IEF run, the IPG strips were incubated in freshly prepared equilibration buffer #1 (50 mm Tris/HCl, pH 8.8, containing 6 M urea, 30% glycerol, 2% SDS, trace amount of bromophenol blue and 10 mg/mL DTT) for 15 min with gentle shaking. The strips were then rinsed in freshly prepared equilibration buffer #2 (50 mm Tris/HCl, pH 8.8, containing 6 M urea, 30% glycerol, 2% SDS, trace amount of bromophenol blue and 45 mg/mL iodoacetamide) for 10 min with gentle shaking. The IPG strips were then rinsed once in the SDS‐gel running buffer before transferred onto the SDS/PAGE (12% SDS‐gel prepared using low florescent glass plates) and sealed with 0.5% (w/v) agarose solution (in SDS‐gel running buffer). The SDS/PAGE was run at 15 °C until the dye front ran out of the gels.

### Image scan and data analysis

Image scans were carried out immediately following the SDS/PAGE using Typhoon TRIO (GE Healthcare) following the manufacturer’s protocols. The scanned images were analyzed by Image QuantTL software (GE Healthcare), and then, in‐gel analysis and cross‐gel analysis was performed using DeCyder software version 6.5 (GE Healthcare). The ratio change of the protein differential expression was obtained from in‐gel DeCyder software analysis.

### Spot picking and trypsin digestion

The spots of interest were picked up using the Ettan Spot Picker (GE Healthcare) based on the in‐gel analysis and spot picking design by DeCyder software. The gel spots were washed several times and digested in‐gel with modified porcine trypsin protease (Trypsin Gold, Promega). The digested tryptic peptides were desalted by Zip‐tip C18 (Millipore). Peptides were eluted from the Zip‐tip with 0.5 μL of matrix solution (α‐cyano‐4‐hydroxycinnamic acid, 5 mg/mL in 50% acetonitrile, 0.1% trifluoroacetic acid, 25 mm ammonium bicarbonate) and spotted on the MALDI plate.

### Mass spectrometry

MALDI‐TOF (MS) and TOF/TOF (tandem MS/MS) were performed on a 5800 mass‐spectrometer (AB Sciex). MALDI‐TOF mass spectra were acquired in reflectron positive ion mode, averaging 2000 laser shots per spectrum. TOF/TOF tandem MS fragmentation spectra were acquired for each sample, averaging 2000 laser shots per fragmentation spectrum on each of the 5–10 most abundant ions present in each sample (excluding trypsin autolytic peptides and other known background ions).

### Database search

Both the resulting peptide mass and the associated fragmentation spectra were submitted to GPS Explorer version 3.5 equipped with the MASCOT search engine (Matrix Science) to search the database of the National Center for Biotechnology Information nonredundant (NCBInr) or Swiss Protein database. Searches were performed without constraining protein molecular weight or isoelectric point, with variable carbamidomethylation of cysteine and oxidation of methionine residues, and with one missed cleavage allowed in the search parameters. Candidates with either protein score CI% or ion CI% > 95 were considered significant.

### Transmission electron microscopy

LNCaP cells cultured for 48 h in the absence or presence of 100 μm hemin were washed 3 times with PBS, fixed in freshly prepared 2.5% glutaraldehyde/formaldehyde in cacodylate buffer for 2 h, and rinsed with 0.2 M cacodylate buffer three times for 10 min each. The cells were then fixed and stained with 1.0% osmium tetroxide in 0.2 M cacodylate buffer for 1 h. After staining, the cells were rinsed with cacodylate buffer three times for 10 min each and then with deionized water twice for 10 min each. The cells were dehydrated through an increasing ethanol series (50% ethanol, 70% ethanol, 90% ethanol, and finally 100% ethanol) for 10 min each time. Embedding resin was prepared by mixing Epon 812, DDSA, NMA, and DMP30 (Electron Microscopy Sciences, Hatfield, PA, USA) as previously described [31]. The cells were transferred to resin:acetonitrile (50:50 mixture) for 1 h to allow resin to penetrate into cells. The resin/acetonitrile mixture was then replaced with 100% resin, which was allowed to infiltrate into cells overnight. The cells and resin were finally transferred to beam capsules and placed in a 55 °C oven for 48 h until the resin hardened. The resin blocks were first trimmed, and then, thin sections (80 nm thickness) of cells were cut using an ultramicrotome (Leica Microanalysis, Buffalo Grove, IL, USA) and placed onto 200‐mesh formvar/carbon‐coated copper transmission electron microscopy (TEM) grids (Electron Microscopy Sciences, Hatfield, PA, USA). The TEM grids were stained with 0.5% uranyl acetate staining to enhance contrast prior to TEM imaging.

### Statistics

Student *t*‐test was used for statistical analysis presenting two‐tail *P* values.

## Results

### Quantification of *M. hyorhinis* by real‐time PCR

**Fig. 1:**
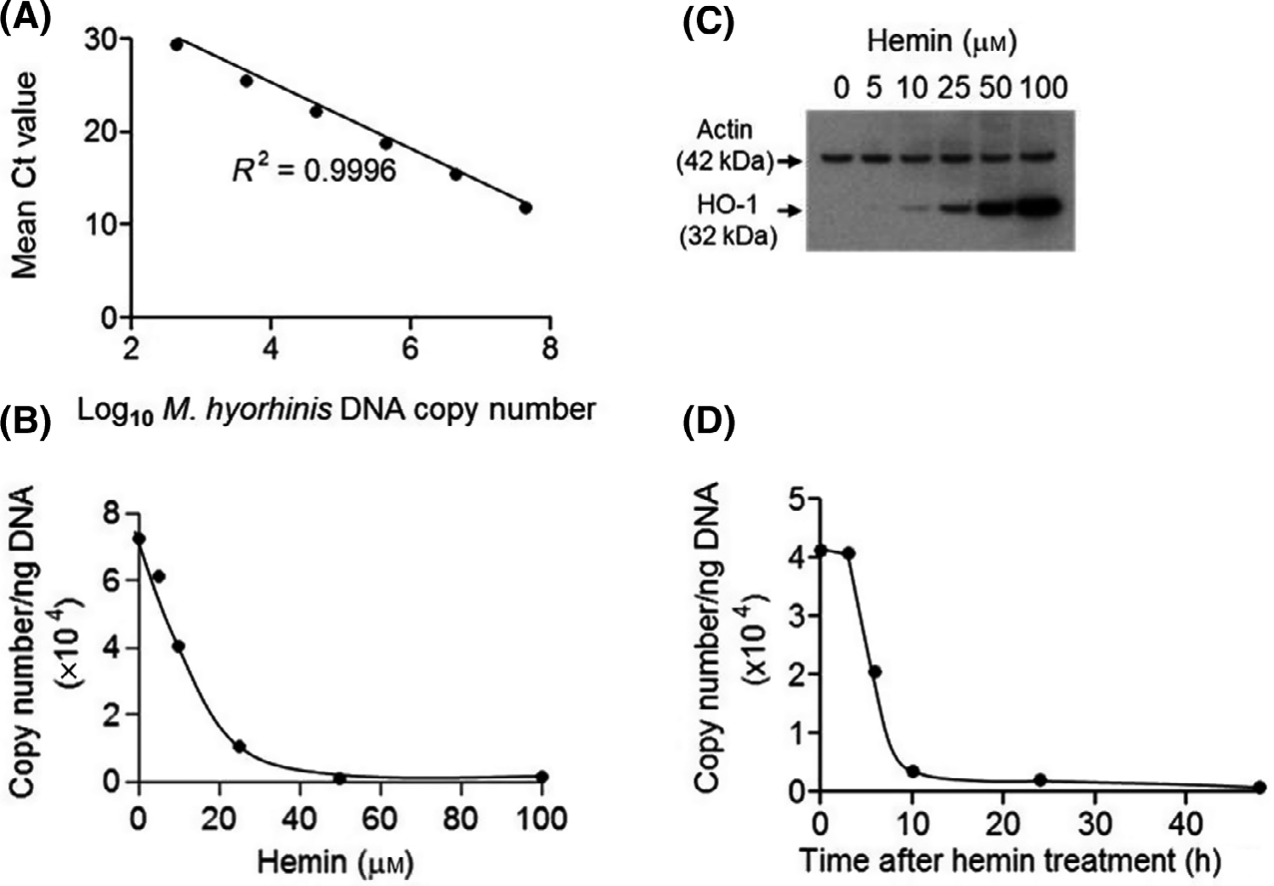
HO‐1 induction inhibits *M. hyorhinis* replication in chronically infected LNCaP cells. (A) Standard curve generated from the Ct values determined by real‐time DNA‐PCR amplification of purified *M. hyorhinis* corresponding to the calculated copy numbers as described in ‘Materials and Methods’. (B) *M. hyorhinis* copy numbers present in the genomic DNA isolated from LNCaP cells cultured for 48 h at the indicated concentrations of hemin. (C) Western blot analysis of hemin‐treated MDM. Cells were incubated with the indicated concentrations of hemin, cellular proteins were separated on an SDS/polyacrylamide gel, transferred to PVDF nitrocellulose membrane, and probed simultaneously with HO‐1 and actin antibodies. The HO‐1 protein (32 kDa) and actin (42 kDa) bands were visualized using the ECL® (enhanced chemiluminescence) detection system (GE Healthcare) and exposed to X‐ray film as described in ‘Materials and Methods’. (D) *M. hyorhinis* copy numbers present in the genomic DNA isolated from LNCaP cells cultured in the presence of 100 μm hemin at the indicated times. The data are representative of two independent experiments.

Using universal mycoplasma primers, we performed real‐time PCR amplification of total DNA isolated from LNCaP cells chronically infected with *M. hyorhinis*. To quantify *M. hyorhinis* by real‐time PCR, we first generated a standard curve by amplifying DNA isolated from the BTS‐7 strain of *M. hyorhinis*. We calculated the copy numbers from the respective DNA molar concentrations. Figure 1A shows a representative *M. hyorhinis* standard curve with linearity between the amplification threshold values (Ct) at varying Log_10_ *M. hyorhinis* copy numbers over a broad linear dynamic range with an R^2^ value of nearly 1.0. We used this standard curve to convert the Ct values into *M. hyorhinis* copy numbers in the infected cells by amplifying known amounts of isolated genomic DNA.

### Hemin treatment induces HO‐1 expression and inhibits *M. hyorhinis* replication in LNCaP prostate cancer cells

Real‐time PCR amplification of the total DNA from *M. hyorhinis* ‐infected LNCaP cells cultured for 48 h in the presence of varying concentrations of hemin revealed a dramatic reduction of cell‐associated *M. hyorhinis* DNA in a dose‐dependent manner, with > 90% inhibition at 100 μm hemin (Fig. 1B). Western blot analysis of total protein isolated from these cells established the induction of HO‐1 in a concentration‐dependent manner without altered expression of the housekeeping protein actin (Fig. 1C). Since hemin‐induced maximal HO‐1 expression at 100 μm hemin without causing cytotoxicity, this concentration was used in subsequent experiments. To determine the earliest time point for reduced *M. hyorhinis* replication, LNCaP cells were cultured in the presence of 100 μm hemin and *M. hyorhinis* was quantified by real‐time PCR of total cellular DNA at the indicated times. As shown in Fig. 1D, hemin treatment profoundly inhibited intracellular *M. hyorhinis* DNA levels within 10 h to nearly undetectable levels after 48 h.

### Effect of penicillin/streptomycin (P/S), gentamicin and hemin on *M. hyorhinis* replication, cell growth and cellular toxicity

**Fig. 2:**
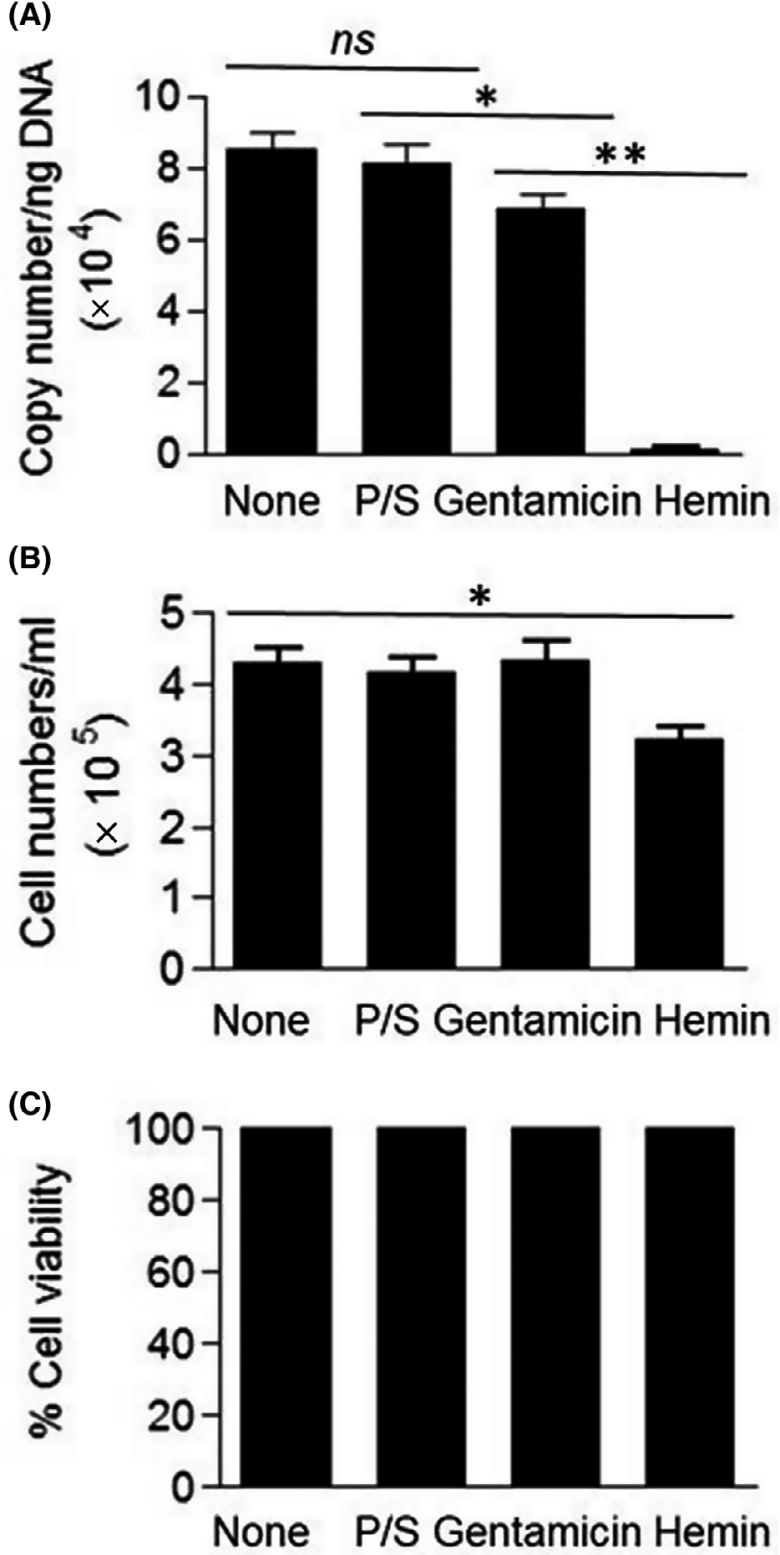
Hemin treatment inhibits antibiotic‐resistant *M. hyorhinis* replication in chronically infected LNCaP cells. LNCaP cells were cultured for 48 h in the absence or presence of penicillin/streptomycin (P/S), gentamicin (10 μg/ml), and hemin (100 μM). (A) Intracellular *M. hyorhinis* DNA was quantified by real‐time PCR as described in ‘Materials and Methods’. (B) Total numbers of cells was determined counted by microscopy. (C) Cell viability was determined by trypan blue exclusion test. Representative data from three independent experiments are presented as mean ± SEM from triplicate observations. ns, not significant; *P = 0.063; **P = 0.0001.

LNCaP cells were cultured at 37 °C for 48 h in the absence or presence of penicillin/ streptomycin and gentamicin at the concentration of 10 μg/mL and 100 μm hemin. The cells were then examined for intracellular *M. hyorhinis* DNA level, cell growth, and cytotoxicity. As shown in Fig. 2A, intracellular *M. hyorhinis* DNA levels in LNCaP cells cultured in the presence of penicillin/streptomycin and gentamicin were similar to those cultured in the absence of these antibiotics with approximately 22% reduction in cells cultured in the presence of gentamicin. However, the level of *M. hyorhinis* DNA in LNCaP cells cultured in the presence of hemin was nearly undetectable. Although hemin treatment partially retarded the growth of LNCaP cells by ˜ 25% (Fig. 2B) and was not cytotoxic (Fig. 2C), it inhibited intracellular *M. hyorhinis* DNA levels by greater than 90% (as shown in Fig. 2A), indicative of a marked reduction of *M. hyorhinis* replication in hemin‐treated LNCaP cells.

### Confirmation of HO‐1‐dependent suppression of *M. hyorhinis* replication

**Fig. 3:**
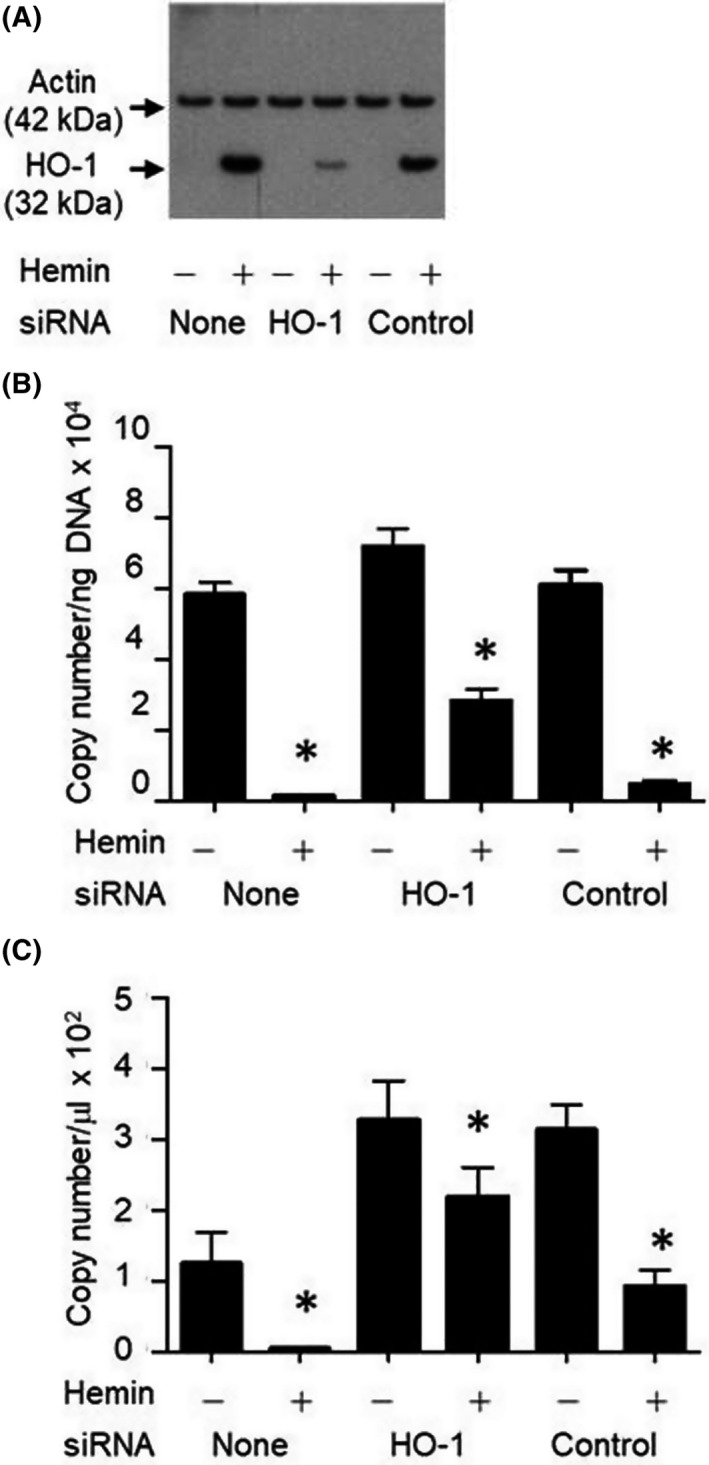
Confirmation of HO‐1‐mediated inhibition of *M. hyorhinis* replication in chronically infected LNCaP cells. (A) LNCaP cells were transfected with control siRNA or HO‐1‐specific siRNA, and hemin‐induced HO‐1 expression (32 kDa) was examined by western blot analysis using actin (42 kDa) as the housekeeping gene control as described in ‘Materials and Methods’. (B) *M. hyorhinis* copy numbers present in the genomic DNA isolated from cells transfected with control siRNA or HO‐1‐specific siRNA cultured for 48 h in the absence or presence of 100 μm hemin. (C) *M. hyorhinis* copy numbers measured in the serum‐free supernatants of cells transfected with control siRNA or HO‐1‐specific siRNA as described in ‘Materials and Methods’. Representative data are presented as mean ± SEM from three experiments. *P < 0.05

To confirm that the reduced *M. hyorhinis* replication is mediated by HO‐1, LNCaP cells were transfected with siRNA specific to HO‐1 prior to culturing in the presence of 100 μm hemin for 48 h. HO‐1 protein expression was determined by western blot analysis, and the level of *M. hyorhinis* DNA was quantified by real‐time PCR. Silencing of HO‐1 substantially reduced its intracellular expression (Fig. 3A), and transfection with HO‐1‐specific siRNA substantially diminished the protective effect of hemin against *M. hyorhinis* infection (Fig. 3B). Transfection of cells with control siRNA did not significantly affect either HO‐1 induction or *M. hyorhinis* replication. To determine the level of cell‐free production of *M. hyorhinis*, cells were first cultured for 24 h in the absence or presence of 100 μm hemin followed by replacing the media with serum‐free media lacking hemin and then culturing cells for an additional 24 h. *M. hyorhinis* DNA was amplified directly from 5 μL of the culture supernatants without DNA extraction. The data from this experiment showed the pattern of cell‐free *M. hyorhinis* copy numbers to be identical to that observed for cell‐associated *M. hyorhinis*, indicating the active production of extracellular mycoplasma by the infected cells (Fig. 3C).

### Proteomic analysis of *M. hyorhinis*‐infected LNCaP cells

**Fig. 4:**
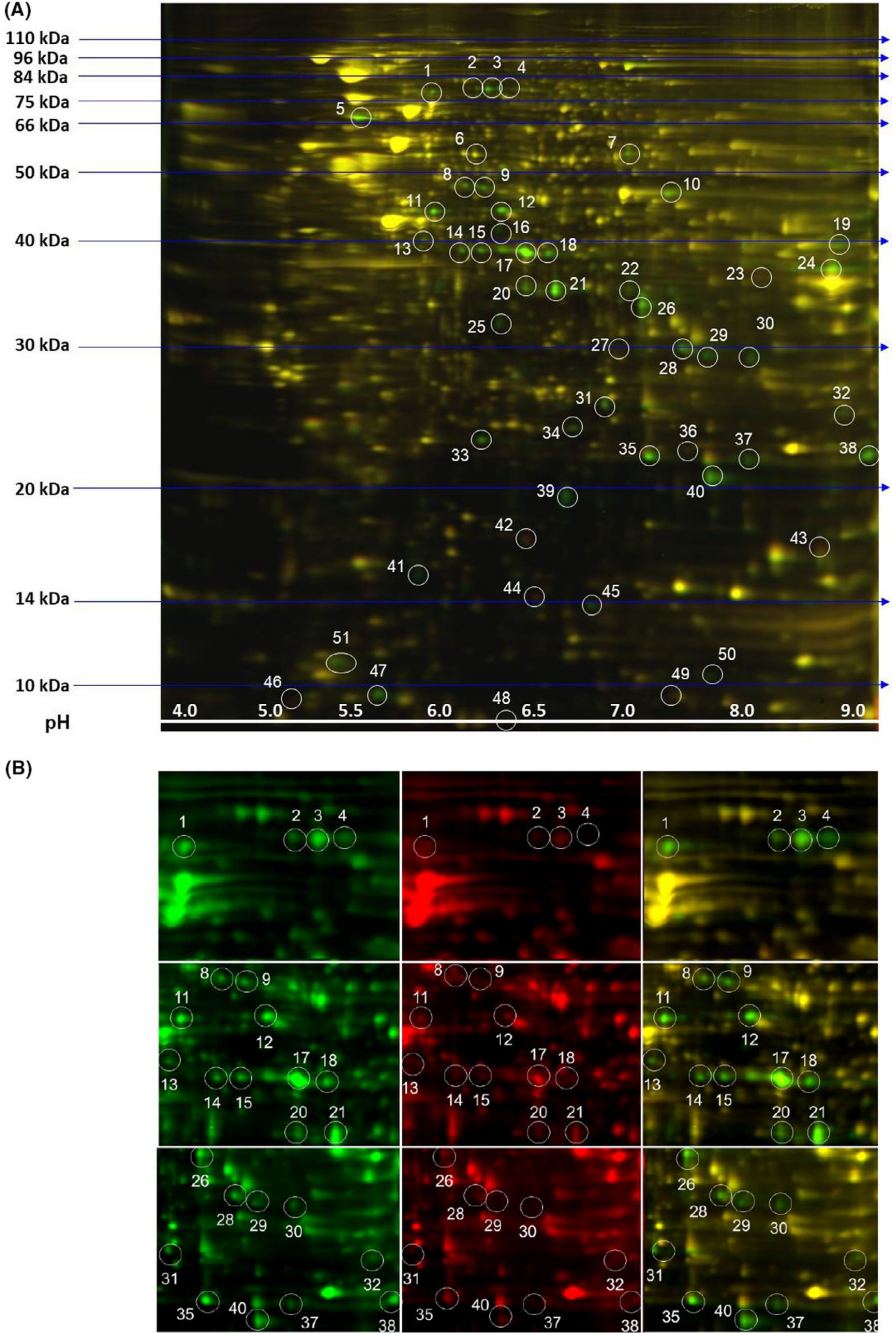
Effect of hemin treatment on *M. hyorhinis* protein expression in chronically infected LNCaP cells. Two‐dimensional proteomic analysis of total proteins isolated from untreated and hemin‐treated *M. hyorhinis* ‐infected LNCaP cells as described in ‘Materials and Methods’. (A) Protein profile of the whole cell lysate isolated from infected LNCaP cells. (B) Differentially regulated proteins isolated from cells cultured in the absence (shown in green) or presence of hemin (shown in red). Unchanged protein expression appears yellow (superimposed green and red).

To determine the proteomic patterns associated with the protective role of hemin against *M. hyorhinis* infection, we performed 2‐D electrophoresis on equal amounts of total protein isolated from untreated *M. hyorhinis*‐infected and hemin‐treated *M. hyorhinis*‐infected LNCaP cells. We labeled the proteins from these cells with Cy3 and Cy5, respectively, and compared protein profiles of hemin‐treated LNCaP cells with the untreated cells to examine for hemin‐induced altered protein expression. We found a number of *M. hyorhinis* proteins present in whole cell lysates isolated from the infected cells cultured in the absence of hemin (shown in green, Fig. 4A). Two proteins (spot numbers 36 and 42) induced by hemin and shown in red were observed at low levels. However, due to their low fluorescence intensities, these spots did not meet the cutoff for mass spectrometry protein identification selection. Figure 4B shows the pattern of differentially regulated cellular proteins in LNCaP cells cultured in the presence of hemin. As shown in this figure, the expression of all *M. hyorhinis* proteins present in the infected cells was dramatically reduced in hemin‐treated cells (shown in red, Fig. 4B). The profile of unchanged protein expression is shown in yellow. The mass spectrometric analysis performed on the differentially regulated proteins revealed that all identified spots represented various *M. hyorhinis* proteins (Table 1).

**Table 1:** Protein identification by mass spectrometry.

Spot No.	Top‐ranked Protein Name (Species)	Accession No.	Protein MW	Protein Score (C.I. %)
1	Translation elongation factor G (Mycoplasma hyorhinis GDL‐1)	gi|378835973	77258	100
3	Dihydrolipoamide dehydrogenase (Mycoplasma hyorhinis GDL‐1)	gi|378835984	67222	100
4	Dihydrolipoamide dehydrogenase (Mycoplasma hyorhinis GDL‐1)	gi|378835984	67222	100
5	Molecular chaperone DnaK (Mycoplasma hyorhinis HUB‐1)	gi|304373393	65004	100
7	Pyruvate kinase (Mycoplasma hyorhinis GDL‐1)	gi|378835975	53045	100
8	Cytosol aminopeptidase pepA (Mycoplasma hyorhinis SK76)	gi|423262589	51031	100
9	Leucyl aminopeptidase (Mycoplasma hyorhinis GDL‐1)	gi|378835535	51003	100
10	Enolase (Mycoplasma hyorhinis HUB‐1)	gi|304373255	48963	100
11	Phosphoglycerate kinase (Mycoplasma hyorhinis GDL‐1)	gi|378835909	44522	100
12	Translation elongation factor Tu (Mycoplasma hyorhinis GDL‐1)	gi|378836033	43818	100
14	Aminopeptidase (Mycoplasma hyorhinis HUB‐1)	gi|304373003	39585	100
15	Pyruvate dehydrogenase E1‐alpha subunit (Mycoplasma hyorhinis MCLD)	gi|385858734	41661	100
17	Pyruvate dehydrogenase E1‐alpha subunit (Mycoplasma hyorhinis MCLD)	gi|385858734	41661	100
18	Pyruvate dehydrogenase E1‐alpha subunit (Mycoplasma hyorhinis MCLD)	gi|385858734	41661	100
19	Glyceraldehyde 3‐phosphate dehydrogenase (Mycoplasma hyorhinis MCLD)	gi|385858603	36544	100
21	Pyruvate dehydrogenase E1 component beta subunit (Mycoplasma hyorhinis HUB‐1)	gi|304373302	35707	100
24	Hypothetical protein MYM_0485 (Mycoplasma hyorhinis GDL‐1)	gi|378835933	39364	100
26	L‐lactate dehydrogenase (Mycoplasma hyorhinis SK76)	gi|423263023	34762	100
28	Fructose‐bisphosphate aldolase class II (Mycoplasma hyorhinis SK76)	gi|423262658	31171	100
31	Probable purine nucleoside phosphorylase transmembrane protein (Mycoplasma hyorhinis HUB‐1)	gi|304373351	25848	100
33	Hexulose 6 phosphate synthase (Mycoplasma hyorhinis HUB‐1)	gi|304373242	25321	100
35	Putative quinone reductase (Mycoplasma hyorhinis GDL‐1)	gi|378835737	23029	100
38	Putative quinone reductase (Mycoplasma hyorhinis GDL‐1)	gi|378835736	22042	100
40	Adenine phosphoribosyltransferase (Mycoplasma hyorhinis HUB‐1)	gi|304373314	20045	100
45	Ribose 5‐phosphate isomerase B (Mycoplasma hyorhinis HUB‐1)	gi|304373420	17031	100
47	Thioredoxin (Mycoplasma hyorhinis GDL‐1)	gi|378835586	13539	100

### Transmission electron microscopy of *M. hyorhinis*‐infected LNCaP cells

**Fig. 5:**
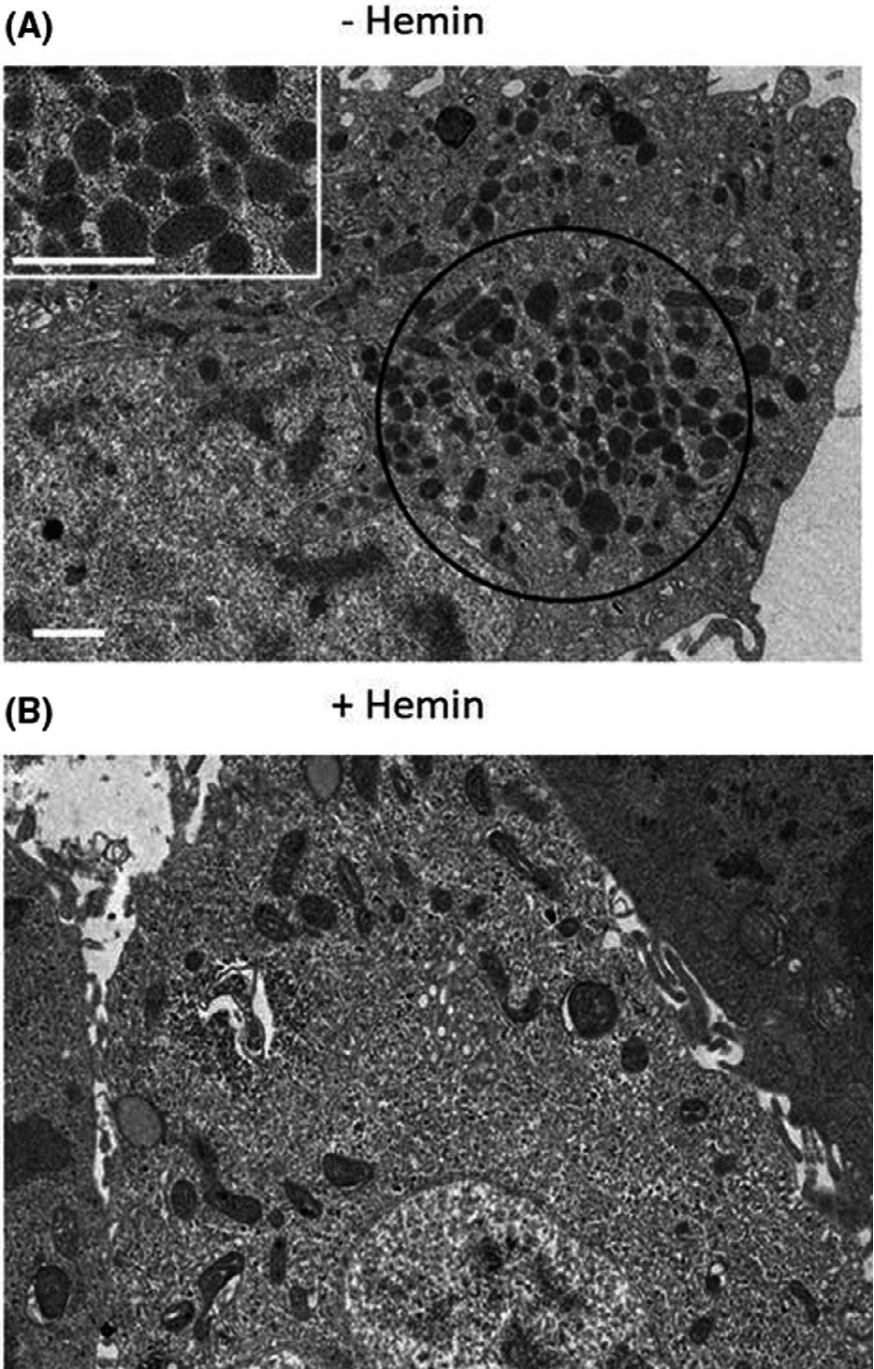
TEM of *M. hyorhinis* ‐infected LNCaP cells cultured for 48 h in the absence (A) or presence of 100 μm hemin (B). Mature microorganism with ovoid to round electron‐dense structures inside of endosomal vacuoles are shown abundantly within the region enclosed by the black circle and at higher magnification in the white inset. Scale bar denotes 1 µm.

Ultrastructural examination of *M. hyorhinis* ‐infected cells revealed a large number of mature microorganisms with ovoid to round electron‐dense structures inside of endosomal vacuoles as shown in the region marked by a black circle and at higher magnification in the white inset of Figure 5A. The diameter of the microorganisms ranged from 0.25 μm to 0.8 μm with an average diameter of approximately 0.36 μm. The observed size of *M. hyorhinis* is consistent with the known size of mycoplasma—spheres of 0.35 μm to 0.50 μm in diameter and rod‐ or dumbbell‐shaped forms up to 1.5 μm in length [32]. Also, the images of infected cells are in agreement with a previously published study by Kornspan *et al*. reporting electron‐dense structures in *M. hyorhinis* ‐infected melanoma cells [17]. Interestingly, such structures were absent from the LNCaP cells cultured for 48 h in the presence of 100 μm hemin (Fig. 5B). The intracellular debris observed in hemin‐treated cells were consistent with inactivated mycoplasma by hemin treatment. These observations are consistent with the hemin‐induced dramatic reduction of *M. hyorhinis* replication as described above and further suggest HO‐1 activated intracellular mediators that disrupt *M. hyorhinis*.

## Discussion

The lack of rigid wall around mycoplasmas, ability to hide inside of cells, and resistance to antibiotics, such as penicillin and streptomycin, often pose extremely difficult challenges to treat chronic mycoplasma infections and attenuate disease progression. In addition, dysregulation of numerous host genes for their survival could further complicate conventional clinical interventions for treating chronic infections [16, 33, 34]. Therefore, new host‐targeted therapeutic approaches, essential for their eradication need to be developed and evaluated.

The involvement of HO‐1 by hemin and other inducers in mediating cellular resistance to a broad range of infections has now been established for more than a decade [19, 20, 21, 22, 23, 24, 25, 26, 27, 28, 29], and more recently also in reducing SARS‐CoV‐2 protein expression in HO‐1‐induced Vero cells [Fig. 2H in Ref. 35]. Specifically, the data shown in Fig. 2H of Olagnier *et al*. [35] showed partial yet significant reversal of the suppressed HO‐1 expression in SARS‐CoV‐2‐infected cells by NRF2 agonists 4‐octyl‐itaconate (4‐OI) and dimethyl fumarate (DMF) while simultaneously inhibiting SARS‐CoV‐2 replication in Vero cells, indicating restoration of the inducible HO‐1‐dependent host protection mechanism. The classical Nrf2‐dependent cellular HO‐1 activation pathway has been known for nearly two decades [36, 37, 38, 39]; therefore, partial restoration of HO‐1 expression by NRF2 agonists 4‐OI and DMF correlating with reduced SARS‐CoV‐2 protein expression, as shown in Fig. 2H of Olagnier *et al*. [35], suggests involvement of HO‐1, at least in part, in an Nrf2‐HO‐1 activation pathway.

While the role of HO‐1 in suppressing virus replication has been widely accepted, its involvement of HO‐1 in regulation of mycoplasma infection of eukaryotic cells has thus far remained unexplored. In the present study, we aimed to determine the involvement of HO‐1 in suppression of *M. hyorhinis* replication in chronically infected prostate cancer cells. We used the physiological HO‐1 inducer hemin in our studies. We found a remarkable reduction of intracellular mycoplasma DNA levels, absence of *M. hyorhinis*‐related proteins by proteomic analysis, and no evidence of intracellular mature microorganism, in hemin‐treated LNCaP cells chronically infected with *M. hyorhinis*.

Our study is the first to our knowledge that demonstrates the HO‐1‐dependent inhibition of *M. hyorhinis* replication in prostate cancer cells, providing evidence for an HO‐1‐mediated cellular protective response as a pivotal host defense mechanism against this cell invading pathogen. Further evidence for the involvement of HO‐1 was confirmed by silencing of HO‐1 gene expression by siRNA transfection which partially yet significantly diminished the protective effect of hemin against *M. hyorhinis* replication. Since HO‐1 protein expression in HO‐1 siRNA‐transfected LNCaP cells was reduced by > 90% compared to a somewhat lower effect on *M. hyorhinis* replication (˜ 30%–50%), possible involvement of HO‐1‐independent or HO‐1 upstream gene regulatory pathways cannot be ruled out, which could be the subject of future studies. Nonetheless, our observations suggest that (a) HO‐1 induction is important for the inhibitory effects on *M. hyorhinis* replication and (b) reduction of HO‐1 expression directly correlates with the increased intracellular mycoplasma level. Our study presents HO‐1 as a key mediator of hemin‐induced host protective effects in LNCaP cells in addition to exhibiting lower cell proliferation. Although the precise postentry events associated with mycoplasma replication or cellular factors contributing to it are not clearly understood, circumventing host defense has been implicated in promoting its pathogenesis [40]. Importantly, numerous studies have documented altered host gene transcription by infectious mycoplasma or by mycoplasma p37 protein for promoting cell invasiveness and metastasis [41, 42, 43, 44, 45, 46, 47, 48, 49]. Therefore, restoring the host defense response may be crucial not only for inactivating the invaded mycoplasmas, or even perhaps interrupting the mycoplasma replication cycle, but also for reducing the severity of the disease pathogenesis.

In summary, our study demonstrates that the induction of cellular HO‐1 protein expression is pivotal for the inhibitory effect of hemin on *M. hyorhinis* replication in chronically infected prostate LNCaP cancer cells. The reduced HO‐1 expression in cells transfected with HO‐1‐specific siRNA proportional to increased *M. hyorhinis* replication provides strong evidence for HO‐1 as a key mediator of hemin‐induced HO‐1 host protective response. Our experimental approach pointing to a putative role for the activation of the innate cellular mechanism in inducing host defense may provide an alternative therapeutic modality to further establish the concept of stimulated innate cellular response against invading pathogens, especially because hemin treatment is FDA‐approved for acute intermittent porphyria. Accordingly, an effective pharmacological stimulation of HO‐1 induction, a protein that is widely distributed in all tissues, may present an attractive therapeutic strategy for overcoming hurdles associated with antibiotic resistance to invading mycoplasmas.

## Conflict of interest

The findings and conclusions in this article are the views of the authors and should not be construed to represent FDA’s view or policies. The authors declare no conflict of interest.

## Author contribution

SD, conceived, designed the study, acquired, analyzed, interpreted the data and wrote the paper; HH, AD, JP, JZ, and YW acquired, analyzed, interpreted the data and edited the paper; VC, PWB and KMY analyzed, interpreted the data, and edited the paper.

## Data Availability

The data that support the findings of this study are available in the figures and tables of this article.
